# Older patients with Hodgkin Lymphoma: Walking the tightrope of efficacy and toxicity

**DOI:** 10.3389/fonc.2022.1017787

**Published:** 2023-01-13

**Authors:** Aisling Barrett, Graham P. Collins

**Affiliations:** Department of Clinical Haematology, Oxford Cancer and Haematology Centre, Churchill Hospital, Oxford, United Kingdom

**Keywords:** hodgkin lymphoma, elderly, chemotherapy, toxicity, anthracycline

## Abstract

Since its initial description, classical Hodgkin lymphoma (cHL) portends a greatly improved prognosis and the goal of treatment in most patients is cure with minimisation of toxicity from treatment. Outcomes in older patients (>60 years old) lag behind those of their younger counterparts however, and cure remains achievable mostly for those who can tolerate full doses of conventional chemotherapy. This review addresses the difference in biology between younger and older patients with cHL and examines the impact of frailty and comorbidities on outcomes. The toxicities of conventional chemotherapy in anthracycline-fit and -unfit patients are examined, with a particular focus on pulmonary toxicity associated with bleomycin in older patients. New advances are discussed, including the possibility of using more targeted therapies such as the anti-CD30 antibody brentuximab vedotin (BV) and checkpoint inhibitors as a method of reducing dependency on conventional chemotherapy for those less well able to tolerate it. Treatment of older patients with cHL remains an area of unmet need in hematological research, and efforts to rectify this knowledge gap should continue.

## Introduction

2022 marks the 190^th^ anniversary of the description of classical Hodgkin lymphoma (cHL) by Thomas Hodgkin in his recognition that six patients with “remarkable enlargement of the absorbent glands” discovered on post-mortem examination suffered from a “primitive affection of those bodies” rather than that resulting from an external source of inflammation or infection (1). This year also marks the 100^th^ anniversary of the attribution to HL that it “presents the most hopeless condition in the whole domain of medicine” (2). It is certain that the prognosis of cHL has dramatically improved in the century since this disheartening statement, but outcomes for patients of >60 years of age (y) continue to lag behind those of a younger cohort (3). This review of cHL diagnosis, prognostication and management in those >60y (hereafter referred to as “older patients”) will attempt to address this issue and highlight new advances for these patients.

## Epidemiology

The incidence of cHL in the United Kingdom is 2.4 cases per 100,000 per year, and it is more common in males than in females (Haematological Malignancy Research Network (HMRN) data). cHL has a characteristic bimodal age distribution with one peak in adolescence and the second in older patients (4). Population studies have shown that approximately 20% patients with cHL are over the age of 60 (5) with an age-specific incidence in the UK of 1.9 per 100,000 per year in the 60-69y age group and 2.18 per 100,000 per year in the group of 70y and older. The incidence of Hodgkin lymphoma in all age groups in the UK has risen by 37% since the early 1990s and by 2035 it is predicted that the incidence will become 4 cases per 100,000 people per year (data from Cancer Research UK). Conversely, data from American patients captured in the Surveillance, Epidemiology and End-Results (SEER) database has shown the incidence has slightly decreased over the same time-period (2.7 cases per 100,000 people in 1992 versus 2.4 per 100,000 in 2019). The reason for this discrepancy between UK and US patients is unclear.

## Clinical features

In many older patients the presentation of cHL is similar to younger patients with lymphadenopathy with or without “B” symptoms (fever above 38C, drenching night sweats, and weight loss of more than 10% of body mass in the previous 6 months) predominating. Some differences in clinical presentation may be observed. In older patients, mediastinal involvement occurs in a quarter of patients (24.6%), with the same number having solely infra-diaphragmatic disease (a disease pattern distinctly uncommon in younger patients) (6).

## Biology

Classical HL (cHL) is a neoplasm of B cells, usually of germinal centre origin, which are identified pathologically by multinucleated Hodgkin/Reed-Sternberg (HRS) cells and a characteristic inflammatory milieu (7). Four pathological subtypes of cHL have been identified, including nodular sclerosis, mixed-cellularity, lymphocyte-depleted and lymphocyte-rich. Mixed-cellularity cHL has a higher predominance in the elderly accounting for 39.5-57.4% of cases with nodular sclerosis accounting for 14.7-50.9% of cases (6, 8). The incidence of lymphocyte-depleted histology in older patients with cHL may be up to 2.5 times higher than in young patients, with poorer outcomes generally seen in these patients without the use of more intensive chemotherapy (3, 9).

The causes of cHL are not fully understood. Epstein-Barr virus (EBV) infection is present in one-third of cHL cases in Western countries and its presence has been implicated in the pathogenesis of the disease (10). In older patients EBV infection correlates with significantly worse overall survival (OS) and disease-specific survival, contrary to a favourable effect on survival seen with cHL patients <15y with EBV infection (11, 12). Detection of EBV DNA is more common in samples from older (>50y) than younger (<15y) cHL patients (71% v 54%, p<0.0001) (13). EBV infection and latent membrane protein (LMP)-1 expression correlates with increased production of cytokines such as macrophage inflammatory protein (MIP)-1α, MIP-1ß and interleukin (IL)-13, which is more common in older patients and equates with poorer prognosis (14). The difference in incidence and outcomes between younger and older patients with EBV-driven cHL is not fully characterised but has been postulated to be due to immunosenescence of older patients which may lead to more rapid tumour growth (12).

EBV infection may have therapeutic relevance; EBV-positive tumours have higher programmed death (PD)-1 expression than EBV-negative tumours and this may render such cases more susceptible to PD-1 receptor inhibition by immune checkpoint inhibitor therapy (15). The magnitude of PD ligand (L)-1 receptor expression by tumour cells has been shown in clinical data to correlate with improved progression-free survival (PFS) following treatment with PD-1 inhibitors, albeit that no specific data exists for older patients (16, 17).

cHL is also more common with human immunodeficiency virus (HIV) infection, and is seen more commonly now in patients with improved immunity in the highly active antiretroviral therapy (HAART) era, a phenomenon thought to be due to an increase in CD4+ T cells leading to immune escape by tumour cells (18).

There is evidence for a genetic contribution to the risk of cHL with a three-fold increase in incidence in cHL in first-degree relatives (19) and one Swedish study showing a new 30% heritability of cHL (20). Genome-wide association studies (GWAS) have shown that polymorphisms in human leukocyte antigen (HLA) genes are implicated in the pathogenesis of cHL, underlying the importance of immune dysregulation in tumorigenesis (21). Most reported cases of cHL with a strong hereditable component are however in young patients. The genetic contribution to the pathogenesis of cHL in older patients has not been fully explored.

The incidence of cHL is also increased in those with autoimmune disease (22). cHL is a rare variant of post-transplant lymphoproliferative disease (PTLD) which is usually EBV-positive and has a poorer outcome than cHL in other settings (23). Patients with PTLD-cHL are older than their *de novo* cHL counterparts, with a median age of 52y versus 36y seen in this study.

## Diagnosis

Excisional biopsy of a lymph node is recommended to confirm the diagnosis of cHL (24). Positron emission tomography (PET) scanning is necessitated in all patients for accurate staging (25), and is preferred over bone marrow biopsy for assessment of bone marrow involvement.

As in younger patients, once diagnosis is confirmed older patients with cHL are categorised into early or advanced stage disease, and early stage disease is further classified by the European Organisation for the Research and Treatment of Cancer (EORTC) or German Hodgkin’s Study Group (GHSG) guidelines as favourable or unfavourable. These allow for treatment stratification as discussed below. The International Prognostic Score (IPS) further allows for prognostic assessment of advanced stage patients which may guide expectations regarding treatment outcomes.

## Age as a prognostic factor

Older age is a recognised poor prognostic factor for cHL (3) (26) with factors such as adverse disease biology, comorbidities and reduced fitness for chemotherapy thought to contribute. Real-world outcomes continue to show worse survival in older patients, with data from 2019 showing a 2-year OS of 65% in patients >60y in comparison to OS rates of >90% in other age cohorts (3). In general, older patients are less likely to be treated at an academic centre, and are far less likely to be enrolled in a clinical trial at diagnosis than their younger counterparts (27). Significantly less radiotherapy (RT) was used in early-stage older versus younger patients (3).

In patients >65y studied in the SEER database, only one-quarter received full regimes of chemotherapy, with the same number receiving no documented treatment (28). Three percent of patients died within one month of diagnosis. Older age, frailty and cardiac comorbidity were significantly associated with increased odds of receiving single-agent chemotherapy or RT alone, with older age retaining this association even after adjustment for frailty and other factors.

Whilst in general clinical trial participation has been shown to positively affect patient outcomes (29), older patients continue to have poorer outcomes than their younger counterparts even in this setting. Patients >60y in the GHSG HD5 to HD9 continued to have worse outcomes than their younger counterparts, with 5-year OS of 65% versus 90% in <60 y (30). Similarly to real-world cohorts the challenge of completion of the full course of chemotherapy predominated, with severe infections and haematological toxicity necessitating dose reduction or interruption in many patients. Fifteen percent of older patients suffered from a grade 3 or 4 infection, in comparison to 6% of younger patients. Patients’ wishes also played a role, accounting for 6% of reasons for premature termination of therapy. Almost 40% of older patients died during the observation period, with 24% dying from adverse events from treatment (acute toxicity, cardiovascular or respiratory effects and secondary malignancy most common). Initial responses to treatment for cHL can be similar in treated younger and older patients, with similar overall response rates (ORR); however relapse is common as 5-year freedom from treatment failure (FFTF) was 60% in older patients compared with 80% in younger patients (p<0.001) (30).

Outcomes in early-stage cHL in the elderly has also been studied (31). A large US-based retrospective study of patients treated between 2004-2012 showed that treatment with a combination of chemo- and RT leads to best outcomes, with OS of 77.7% after median follow-up of 40.4 months versus 58.1% for patients receiving chemotherapy alone and 54% for those receiving solely RT (p<0.0001), indicating that outcomes are best for patients fit enough to receive combined modality therapy.

Relapse following first line treatment for cHL in an older cohort portended a poor prognosis in the era before targeted therapies (32). Although 69% of patients in this study published in 2013 were treated with aggressive strategies based on traditional chemotherapy, median OS for the entire cohort was only 12 months, with high-risk patients as defined by anaemia, early relapse and advanced clinical stage generally unsalvageable by any type of therapy (32).

## Frailty and comorbidities as prognostic factor

Comorbidities and frailty contribute to poorer outcomes in older patients with cHL, and attempts have been made to build prognostic scores which allow for improved outcome prediction. Geriatric assessments are recommended in older patients with haematological malignancy to screen for frailty, and poor scores have been shown to predict shorter OS as well as higher rates of treatment non-completion and adverse effects from treatment (33, 34).

The Cumulative Illness Rating Scale (CIRS) has been studied in older cHL patients. In one study, half of patients had at least one severe comorbidity as defined by the scale (35). Interestingly, patients with a severe comorbidity were more likely to have advanced-stage disease (p=0.003), and predictably were less likely to receive anthracycline-containing chemotherapy. The probability of OS at 3 years was 88% (95% CI: 71-95%) in patients without versus 46% (95% CI: 29-62%) with severe comorbidities, with the same effect seen when comparing all patients who received anthracycline-containing chemotherapy, suggesting that frailty contributes to worse outcomes even in those deemed fit enough for intensive treatment. Having said that, it is clear that outcomes are generally improved when conventional chemotherapy can be delivered effectively to older patients (36). The CIRS-geriatric score was also studied in older patients in American academic centres, where activity of daily living (ADL) loss was predictive of reduced PFS and OS, and CIRS ≥ 10 was associated with early treatment discontinuation due to toxicity (37).

A putative prediction model for 1-year mortality in older adults with cHL has been developed based on analysis of more than a thousand older patients, and includes age, Charlson comorbidity index (CCI), B symptoms and advanced stage at diagnosis (38). These patients all received aggressive chemotherapy and therefore this model may be useful in prediction of outcomes for patients considered sufficiently fit for full treatment.

## Treatment

### Fit for anthracycline

As can be inferred from the data presented above, the decision of whether a patient is suitable for conventional chemotherapy for cHL may have a profound impact on an older adult’s likelihood of survival following diagnosis. Geriatric assessments will help stratify decision-making, and all patients considered for anthracycline-based therapy should undergo cardiac assessment by way of echocardiography, with pulmonary function testing (PFT) required prior to bleomycin therapy if clinically indicated.

ABVD chemotherapy (doxorubicin, bleomycin, vincristine and dacarbazine) is an established standard of care for cHL management. 9.6% of patients included in the RATHL trial examining the regimen’s use in a PET-directed fashion in advanced cHL patients were aged ≥ 60y, with the oldest patient on trial aged 79y (39). Undoubtedly ABVD is a more toxic treatment in older patients, and bleomycin-related lung toxicity is more common in older cHL patients (40). In a French study of 147 patients >60y with cHL, bleomycin-related toxicity necessitated reduction or removal of the drug from the treatment plan of over a third of patients (41). The median number of cycles of full ABVD received was 6 (1–8). One-third of patients studied died within a median follow-up of 58 months and 14% of these deaths were due to pulmonary toxicity. In another study, one-quarter of all older patients treated with ABVD or the Stanford V regimen (doxorubicin, vinblastine, mechlorethamine, vincristine, bleomycin, etoposide, prednisone) developed lung toxicity (42). As in younger patients, use of granulocyte colony-stimulating factor (G-CSF) is linked to an increase in pulmonary complications when used with bleomycin-containing regimens (43). As part of their HD10 and HD13 trials, the GHSG study group randomised older early-stage favourable patients to either 2 or 4 cycles of ABVD or 2 cycles of AVD followed by involved-field RT (IF-RT) (44). PFS and OS outcomes were similar among the groups but there was considerable additional toxicity and mortality with 4 cycles of ABVD, leading the authors to conclude that there are unacceptably high rates of adverse events with 4 cycles of ABVD in the context of early-stage favourable cHL in older patients. It is therefore generally advisable that if ABVD is used in older patients, no more than 2 cycles should contain bleomycin.

The HD13 study examined whether dacarbazine or bleomycin could be safely omitted in early-stage favourable patients (45). Omission of dacarbazine from the ABVD regimen was shown to be inferior to the full regimen and AVD could not be shown to be non-inferior to ABVD with respect to FFTF, with late relapses (relapse >1 year after treatment cessation) increased in the AVD group (6.5% versus 3.5% with ABVD). All patients in HD13 received radiotherapy and it is likely that when solely relying on chemotherapy such as for advanced stage patients, omission of bleomycin may have a more profound effect. Therefore whilst omission of bleomycin maybe indicated in patients with additional risk factors for bleomycin toxicity (e.g. renal impairment, prior mediastinal irradiation), it is likely to be associated with increases in relapse rate. It is the authors’ view that in the 60-70y group with no other risk factors for bleomycin pulmonary toxicity, it is reasonable to use a maximum of 2 cycles of ABVD with further cycles of AVD being administered if required. The decision to include bleomycin needs to be individualised and if it is given careful clinical monitoring after each cycle is advised with prompt cessation if features of pulmonary toxicity occur.

BEACOPP (cyclophosphamide, doxorubicin, etoposide, vincristine, bleomycin, procarbazine and prednisolone) has been studied in older patients and has been found to be unacceptably toxic, with a fifth of patients dying from adverse effects of chemotherapy (46); similarly the BACOPP regimen (etoposide omitted) had a 10% mortality rate when studied in older patients (47).

Interim PET scanning performed after 2 cycles of conventional chemotherapy is standard of care in younger patients with cHL as mentioned previously, and this approach has been validated in older patients (48, 49). These two studies confirm that interim PET positivity leads to poorer PFS and OS outcomes and reaffirm that interim PET should be performed in older patients with cHL as a means of prognostication and guidance of subsequent radiotherapy.

The anti-CD30 antibody-drug conjugate brentuximab vedotin (BV) is licensed in the US in combination with chemotherapy for upfront treatment of advanced cHL and in Europe in combination with AVD for stage IV disease. This was based on the ECHELON-1 trial which demonstrated a significantly improved modified PFS with BV-AVD compared with ABVD (50) and on longer follow up, a small but significant OS difference is now evident. A subgroup analysis of 186 ECHELON-1 patients >60y with a median age of 67y showed a trend towards improvement in 5-year PFS in the BV+AVD arm which was not significant (67.1% versus 61.6%, p=0.443). There was a different side effect profile in the BV + AVD arm, with higher rates of peripheral neuropathy (PN) and febrile neutropenia (FN) but less pulmonary toxicity, as may have been expected with the omission of bleomycin. Indeed, 3 out of the 5 deaths on the ABVD arm were due to pulmonary toxicity underscoring the increased risk of bleomycin use in older patients. This remains an attractive bleomycin-free regimen due to outcomes in older patients at least as good as seen with ABVD.

In an effort to make this combination more tolerable for older patients, sequential BV and AVD therapy has been examined in a phase II study for upfront cHL treatment in older patients with advanced stage disease (51). Two cycles of BV were followed by four cycles of AVD with four further cycles of BV. The CR rate was an impressive 93% with a 2-year OS of 93% (95% CI: 80-98%), indicating promise for this type of treatment as a method of achieving cure in these patients. CIRS-geriatric scores were calculated for patients on-study and scores of ≥ 10 were associated with inferior PFS (45% at 2 years versus 100%, p<0.001), indicating that frailty is still indicative of worse outcomes even with targeted therapies.

Data regarding other anthracycline-containing regimens for older patients with cHL are available. CHOP (cyclophosphamide, doxorubicin, vincristine, prednisolone) with or without RT was evaluated in 29 older patients with cHL, with a complete response (CR) rate of 93% but with early relapse (<2 years) occurring in 5 (17.2%) of patients (52). A subsequent study using the Swedish database however compared the outcomes of CHOP with ABVD and ABVD was associated with superior outcomes (53). A phase II clinical trial has examined the use of the novel PVAG regimen (prednisolone, vinblastine, doxorubicin, and gemcitabine) in older patients with nearly 80% of patients responding with CR and 3-year OS rates of 66% (95% CI: 50-78%) but with a majority of patients suffering a grade 3 or 4 toxicity due to treatment (54).

VEPEMB (vinblastine, cyclophosphamide, procarbazine, etoposide, mitoxantrone and bleomycin) is an alternative treatment schedule for older patients with cHL, which was evaluated as part of the UK-based Study of Hodgkin in the Elderly/Lymphoma Database (SHIELD) study which incorporated a comorbidity assessment tool to examine for the effect of frailty on outcomes (55). A subsequent phase III randomised trial compared VEPEMB with ABVD in older but not frail patients (56). With a median observation period of 76 months, 5-year PFS and OS were non-significantly better in the ABVD group, but there were 4 (7.4%) occurrences of grade 4 cardiac or lung toxicity with ABVD use versus none in the VEPEMB arm. Due to this signal for reduced efficacy, this regimen has fallen from favour for routine use in older cHL patients.

The ACOPP regimen modifies BEACOPP with omission of bleomycin and etoposide and dose reduction of cyclophosphamide, and has been developed for use in older cHL patients (57). A small number of older patients have been treated with this regimen with all attaining CMR and with treatment relatively well tolerated, indicating that this regimen deserves further study.

Other strategies have been employed in an attempt to bridge the gap between improvements in treatment efficacy and reduction of adverse events. A recent phase I trial examined the use of lenalidomide in combination with AVD in older patients with cHL (58). The recommended phase II dose of lenalidomide was 20mg with an ORR of 86% at this dose; the 3-year estimate for PFS was 69.7% (95% CI: 50.3-89.1%) and OS 83.8% (69.3-98.4%). However, hematological toxicity was frequent, and the authors suggested that AVD may not be the best backbone for addition of lenalidomide in older patients.

Liposomal doxorubicin may be another option especially for patients with cardiac comorbidities, and one which has been studied in older patients as well as younger patients with cardiac issues in upfront cHL treatment (59). Its use with the traditional backbone of bleomycin, vincristine and dacarbazine led to CR rates of 77% (95% CI: 62-88%) with 3-year OS and PFS estimates of 70% and 43% respectively, with grade 3-5 cardiac events in just 4.2% of patients.

Thus among chemotherapy regimens, ABVD or, if reimbursed, AVD combined with BV (concurrently or sequentially) emerge as standards of care albeit with no more than 2 cycles of bleomycin tolerated by older patients. Indeed, when BV is not available, many centres simply omit the bleomycin and treat with AVD in patients over 60y accepting a small increase in risk of relapse.

### Unfit for anthracycline

A number of strategies have been undertaken to try to abrogate the dismal prognosis experienced by those who are not fit for conventional chemotherapy. It is recommended that in these patients, some form of non-intense therapy should be attempted as quality of life improvements may often be achieved even if cure is not likely. CVP/CEP (chlorambucil, vinblastine, procarbazine, prednisolone, cyclophosphamide, etoposide and bleomycin) and VBM (vinblastine, bleomycin and methotrexate) treatments have been historically studied in this cohort, with varying effectiveness (60, 61). The ChlVPP regimen (chlorambucil, vincristine, procarbazine and prednisolone) has also been used, but addition of doxorubicin and bleomycin to the regimen led to significantly improved OS (30% at 5 years versus 67%, p=0.0086), which may not be well tolerated in frail elderly patients (62).

BV monotherapy has been studied as sole upfront treatment for older patients with cHL who were unfit or ineligible for conventional chemotherapy (63). Baseline geriatric assessments were performed in these patients, with four-fifths of patients impaired in at least one aspect of testing and half of patients having significant comorbidities. The ORR was 92%, with 73% of patients attaining CR, and the median PFS was 10.9 months (2.6-22.3). 30% developed peripheral neuropathy because of BV use. BV monotherapy was also examined in patients deemed unfit for combination chemotherapy as part of the BREVITY trial, where the CMR rate after 4 cycles was 25.8%, median PFS was 7.3 months (95% CI: 5.2–9.0%) and median OS was 19.5 months, leading the authors to conclude that BV alone was a suboptimal treatment even for unfit patients (64).

Dacarbazine or bendamustine have been added to BV to attempt to improve outcomes for older frail patients who would not tolerate conventional chemotherapy (65). BV in combination with bendamustine is considerably toxic, with serious adverse events in 65% of patients on the study and 2 deaths, leading to discontinuation and premature closure of enrolment for this arm of the study. BV with dacarbazine led to a CR rate of 62% and median PFS of 17.9 months, but again with considerable toxicity necessitating early discontinuation of treatment in over half of patients. Geriatric assessments were performed in this study which highlighted the significant comorbidity burden and frailty of many cHL patients.

The immune checkpoint inhibitors nivolumab and pembrolizumab are approved for treatment of relapsed or refractory cHL based on impressive efficacy and durability of responses when used as monotherapy (66, 67). Their efficacy alone and in combination with chemotherapy and their unique side effect profile which does not overlap with conventional chemotherapy raises the intriguing possibility of use in older cHL patients to reduce the reliance on traditional measures to control disease. To this end, nivolumab monotherapy has been trialled in patients over 60y with co-morbidities defined as a CIRS-G score of 6 or more (68). All patients received nivolumab monotherapy, with the addition of vinblastine if the PET scan after 12 weeks showed active disease. 28.6% of patients achieved a CMR to nivolumab alone and the median PFS was 9.8 months (with 20.1 months median follow-up). Concerningly, 23.4% of patients died during treatment, 2/64 from treatment toxicity and 6/64 from lymphoma. BV in combination with nivolumab has shown to be highly active in predominantly younger patients with relapsed disease (69). The combination has been examined in upfront use in older cHL patients (70). The ORR was 64% which was less than the prespecified activity criteria of 80% so the study was closed early. The median PFS was not reached in the 52% of patients who achieved CMR and was 18.3 months in the overall cohort. Just under half of patients developed peripheral neuropathy and one patient died from cardiac arrest which may have been treatment-related.

Single-agent palliative treatments may have to be considered in very frail or elderly patients and there are published data regarding some different approaches. Vinblastine monotherapy was used in a small number of patients with a median age of 85y with an ORR of 45% and a median OS of 33 months which may likely have been in part due to clinical improvement following vinblastine administration enabling subsequent initiation of multi-agent chemotherapy, emphasising the clinical benefit that can be derived from chemotherapy use in even our frailest patients (71). First-line RT alone for disease control in all-comers with cHL (22% were >/= 60y) led to impressive CR rates of 88%, with 42% of patients relapsing within a median time of 21 months; this study is illustrative of the potential for long-term remissions in the small number of patients where any form of chemotherapy cannot be tolerated (72).

Upfront treatments in older patients with cHL which have been studied as part of a prospective clinical trial are summarised in **Table 1**.

**Table 1:** Comparison of prospective trials regarding upfront treatment of older patients with classical Hodgkin lymphoma (CHL).

Reference	Age in years (range)	Number of patients	Experimental arm	Control arm	Efficacy outcomes
Levis et al., 2004 (73)	71 (66–83)	105	VEPEMB	None (phase II)	- CR in 76% - 5-year OS of 64%
Ballova et al., 2005 (46)	69.5 (66–75)	68	BEACOPP	COPP-ABVD	- CR in 76% - 5-year OS of 50%
Halbsguth et al., 2010 (47)	66.7 (60-75)	65	BACOPP	None (phase II)	- CR in 85% - 3-year PFS of 60% - 3-year OS of 71%
Böll et al., 2011 (54)	68 (60-75)	59	PVAG	None (phase II)	- CR in 78% - 3-year OS of 66% - 3-year PFS of 58%
Proctor et al., 2012 (55)	73 (61-85)	103 - Frail patients excluded by GA	VEPEMB	None (phase II)	- CR in early stage in 74%, advanced stage 61% - 3-year OS in early stage of 81%, advanced stage 66% - 3-year PFS of 74%, advanced stage 58%
Evens et al., 2013 (42)	65 (60-83)	44	ABVD	Stanford V	- CR in 64% - 5-year OS of 58%
Böll et al., 2013 (32) *	65 (60-75)	117 - Early stage only	ABVD (4 cycles)	30 or 20 Gy of RT was randomis-ation	- CR in 89% - 5-year OS of 81% - 5-year PFS of 75%
Forero-Torres et al., 2015 (63)	78 (64-92)	27 - Patients were ineligible for conventional chemotherapy	BV	None (phase II)	- CR in 73% - Median PFS of 10.9 months (2.6-22.3)
Zallio et al., 2016 (56)	72 (65-80)	54 - Frail patients excluded by GA	VEPEMB	ABVD	- 5-year PFS of 48% vs. 70% (not significant) - 5-year OS of 63% vs. 77% (not significant)
Böll et al., 2016 (44) *	65 (60-75)	287 - Early stage cHL only	ABVD	2 cycles of ABVD or AVD each followed by IFRT or 4 cycles ABVD + IFRT	- CR in 88-99% - 5-year PFS of 78-79% - 5-year OS of 84-91%
Friedberg et al., 2017 (65)	69- 75 (62-88)	42	BV + bendamustine	BV + dacarbazine (phase II, technically)	- BV + dacarbazine: CR in 62% and median PFS of 17.9 months - BV + bendamustine: CR in 88% and median PFS not reached
Evens et al., 2018 (51)	69 (60-88)	48	BV X2 -> AVD X6 -> BV X4	None (phase II)	- CR in 93% - 2-year PFS of 84% - 2-year OS of 93%
Böll et al., 2019 (58)	25 (61-76)	68	Lenalidomide + AVD	None (phase I)	- CR in 76% - 3-year PFS of 69.7% - 3-year OS of 83.8%
Salvi et al., 2019 (59)	75 (46-84)	47	MBVD	None (phase II)	- CR in 77% - 3-year OS of 70% - 3-year PFS of 43%
Cheson et al., 2020 (70)	71.5 (64-77)	46	BV + nivolumab	None (phase II)	- CR in 48% - Median PFS of 18.3 months
Gibb et al., 2021 (64)	77 (69-82)	31	BV	None (phase II)	- CR in 25.8% - Median PFS of 7.3 months - Median OS of 19.5 months
Lazarovici et al., 2021 (68)	75 (62-91)	56	Nivolumab +/- vinblastine	None (phase II)	- CMR in 28.6% - Median PFS of 9.8 months
Evens et al., 2022 (74) **	68 (60-83)	186	BV + AVD	ABVD	- CR in 61% - Modified PFS of 61.6-67.1%

*= these two trials have overlapping trial populations.

**= sub-study of ECHELON-1.

VEPEMB, vinblastine, cyclophosphamide, procarbazine, etoposide, mitoxantrone and bleomycin. CR, complete response; OS, overall survival; BEACOPP, cyclophosphamide; doxorubicin, etoposide, vincristine, bleomycin, procarbazine, prednisolone; ABVD, doxorubicin, bleomycin, vincristine and dacarbazine; BACOPP, bleomycin, cyclophosphamide, doxorubicin, vincristine, procarbazine, prednisolone. PFS, progression-free survival; PVAG, prednisolone, vinblastine, doxorubicin, gemcitabine; CRu, complete remission/unconfirmed; GA, geriatric assessment; TRM, treatment-related mortality; Stanford V, doxorubicin, vinblastine, mechlorethamine, vincristine, bleomycin, etoposide, prednisone; FFS, failure-free survival; RT, radiotherapy; PN, peripheral neuropathy; IFRT, involved-field RT; BV, brentuximab vedotin; EOT, end of treatment; ORR, overall response rate; MBVD, liposomal doxorubicin, bleomycin, vincristine and dacarbazine; PN, peripheral neuropathy; PET, positron emission tomography; CMR, complete metabolic response; FN, febrile neutropenia.

### Relapsed/refractory disease

The current standard of care in younger patients with relapsed or refractory cHL at present includes use of high-dose chemotherapy (HDT) with autologous stem cell transplant (ASCT) conditioned with BEAM/LEAM (carmustine/lomustine, etoposide, cytarabine, melphalan) based on two randomised trials albeit without a demonstrable overall survival benefit (75, 76). Typical first-line relapse regimens include GDP (gemcitabine, cisplatin, prednisolone), ESHAP (etoposide, cisplatin, cytarabine), ICE (ifosfamide, etoposide, carboplatin) and DHAP (dexamethasone, cisplatin, cytarabine),. This remains a valid option in patients aged >60y provided they are sufficiently fit for ASCT. Two recent retrospective studies have looked at the feasibility of this approach in older patients. In a French cohort, 6.6% of older patients died within 3 months of ASCT from toxic events including organ failure and pneumonia (77). With a median follow-up of 54 months, 5-year estimates of OS and PFS for the entire group were 67 and 54%, respectively. Another recent study showed that toxicity following ASCT was comparable between patients 50-59y and >60y, and that poorer outcomes were associated with disease status at time of ASCT and higher comorbidity scores, but not age (78). Of note, secondary malignancies after ASCT are more common in older than younger cHL patients, with one study finding an incidence of 22% of secondary cancers (not including superficial skin cancers) in patients >55y (79). This study indicates that HDT and ASCT is a feasible treatment for fit older cHL patients with relapsed or refractory disease.

Management at further relapse and for patients not sufficiently fit for this approach remains extremely challenging, with a paucity of data to influence options. Novel agents have been used in this setting in older patients. BV monotherapy has been examined in a cohort of older patients unfit for conventional approaches at first relapse or in the primary refractory setting as part of the FIL ONLUS trial (80). Just under one-quarter of patients attained CR after treatment and the median PFS was 8.8 months suggesting useful efficacy. Of note, only 12% finished the total duration of chemotherapy and one-third of patients developed PN, re-affirming that even BV alone is not without adverse effects.

Everolimus, a mammalian target of rapamycin (mTOR) inhibitor, has led to ORR of 45.6% in relapsed cHL patients of all ages with a median of 4 prior lines of therapy (81). Cytopenias, stomatitis and rash predominated as adverse effects and 61.4% of patients required dose reduction due to toxicity. Combination of everolimus or another mTOR inhibitor sirolimus with the histone deacetylase (HDAC) inhibitor vorinostat has also been examined as part of a phase I dose escalation study and showed activity, with ORR of 55% with sirolimus and 33% with everolimus, albeit in the pre-checkpoint inhibitor era (82).

PD-1 inhibitors nivolumab and pembrolizumab are licensed for use in relapsed and refractory cHL patients, with patients aged >65y accounting for 4% of patients in the seminal trial evaluating nivolumab and 8.6% for pembrolizumab in this setting (83, 84). PD-1 inhibitors have a contrasting side effect profile and the main concern with their use is of immune-related adverse drug reactions (irADRs) such as colitis, thyroiditis or liver abnormalities (85). A study of adverse events specifically in an older cohort of patients receiving PD-1 inhibitors for any indication revealed an incidence of 53.9% for cutaneous irADRs, 20.3% for thyroiditis and 17.5% for colitis, with a 6.5% hospitalisation rate; however the frequency of adverse events was not higher than in younger patients (86). Whether this remains the case for older patients specifically with Hodgkin lymphoma is uncertain.

The question of which novel agent to use in relapsed cHL has been addressed in KEYNOTE-204 which is a phase III study comparing BV and pembrolizumab in patients ineligible for or relapsed after ASCT. 14% of patients in the BV group and 18% of patients in the pembrolizumab group were >65y. After a median observation period of 25.7 months, median PFS was 13.2 months (95% CI: 10.9–19.4%) for pembrolizumab versus 8·3 months (5·7–8·8) for BV, with pneumonitis more common in the pembrolizumab group but neutropenia and PN seen more commonly in the BV group. The PFS differential did not reach statistical significance in the older cohort specifically but this was an underpowered subgroup analysis, indicating that further study in older patients is warranted. Applying the rationale of using the more effective treatments earlier in the treatment pathway, this would suggest using a PD-1 inhibitor before BV although other considerations may lead to an alternative strategy.

## Future directions

It is clear that the outcome of older, and in particular frailer, patients with cHL is poor compared with a younger, fitter cohort. The challenge is to maintain and even improve survival outcomes, whilst reducing the chemotherapy burden for patients. Indeed with most evidence pointing to reduced treatment delivery leading to worse outcomes, the two aims go hand in hand. One suggested approach in frontline treatment could be to add additional agents to the backbone of traditional chemotherapy to enable reduction of the doses or number of cycles of treatment, reducing myelosuppression and other forms of toxicity. Intriguingly, several reports have suggested that treatment with PD-1 inhibitors can re-sensitise cHL to standard chemotherapy and thus act as a bridge to autologous or allogeneic transplantation in the relapsed or refractory setting (87). It is possible that this approach may be valid in the upfront setting. Initial data from trials treating mainly younger patients with a checkpoint inhibitor prior to standard chemotherapy have shown impressive durable remission rates (16, 88, 89). The issue with these studies is that although they omit bleomycin, they still use the standard number of chemotherapy cycles which is unlikely to reduce the toxicity for older patients, particularly as the bleomycin is replaced with a checkpoint inhibitor which will bring its own toxicity profile. Trials are needed to explore whether incorporating checkpoint inhibition into the frontline treatment of cHL can enable a reduction in chemotherapy burden whilst maintaining efficacy, especially for older patients less likely to tolerate chemotherapy.

Immune checkpoint-blocking therapies have thus far focussed on PD-1 inhibition, which has been successful in treatment of cHL. Other molecules involved in this pathway include lymphocyte activation gene (LAG)-3 and T-cell immunoglobulin and mucin-domain containing 3 (TIM-3) which is expressed on different T regulatory cells than PD-1 and thus hypothetically could be used in combination with anti-PD-1 therapy in cHL to promote effective tumour infiltration (90).

Combining checkpoint inhibitors with traditional chemotherapy may improve on results with PD-1 inhibition alone in the relapsed or refractory setting, and studies examining the use of pembrolizumab in combination with bendamustine (clinicaltrials.gov identifier: NCT04510636) and azacytidine (clinicaltrials.gov identifier: NCT05355051) are currently recruiting in older adults.

Chimeric antigen receptor (CAR) T- cell therapy has become established in B cell malignancies, and attempts to use CD-30-directed CAR-T cells have been successful in relapsed and refractory cHL (91). Patients in this study ranged up to a maximum age of 69y and were heavily pre-treated, with a median of 7 prior lines of therapy. Of those who received fludarabine-based conditioning, 59% achieved CR and there was a 1-year PFS and OS of 36% (95% CI: 21-51%) and 94% (95% CI: 79-99%) respectively. CAR-T has well-described side effects of cytokine release syndrome (CRS) and neurotoxicity, however in this study only grade 1 CRS was seen and no neurotoxicity was described, suggesting that CAR-T may be a valid treatment option in fit older cHL patients.

## Conclusion

CHL is often regarded as a success story of modern medicine with improvement in treatments transforming a universally fatal disease into a cancer which is highly curable in most patients. This paradigm is indeed true and should be celebrated. However, there are clearly areas of unmet need and older patients represent a considerable challenge to treatment both in the frontline setting and at relapse. ABVD (normally with no more than 2 cycles containing bleomycin and in some patients with omission of bleomycin entirely) and AVD combined with BV can be considered standard for patients fit to receive chemotherapy albeit with inferior outcomes compared to younger patients. Bleomycin- and anthracycline-containing regimens may not be suitable for a minority of frail older patients due to toxicity and alternative regimens are often associated with other significant toxicities. New approaches are required which will likely incorporate active agents with different toxicity profiles. A continued understanding of the biology of this fascinating disease along with a better understanding of baseline risk will also enable a more tailored and hopefully less toxic treatment approach in the future.

## Author contributions

AB co-wrote the manuscript. GC devised the concept, co-wrote and edited the manuscript. All authors contributed to the article and approved the submitted version.
